# *Dendrobaena veneta* avoids ethyl pentanoate and ethyl hexanoate, two compounds produced by the soil fungus *Geotrichum candidum*

**DOI:** 10.7717/peerj.12148

**Published:** 2021-09-08

**Authors:** Eileen M.S. Reed, Mariel O. O’Connor, Ione C. Johnson, Wayne L. Silver, Cecil J. Saunders

**Affiliations:** 1 Department of Biology, Wake Forest University, Winston-Salem, NC, United States of America; 2 South Stokes High School, Walnut Cove, NC, United States of America

**Keywords:** Avoidance behavior, Chemosensory, Chemical Senses, Aversion, T-maze, *Eisenia hortensis*, Dendrobaena veneta

## Abstract

Earthworms shape the biological and physicochemical qualities of the soil they choose to reside in, but our understanding of the specific chemicals that attract or repel a particular species of earthworm remains incomplete. Current research indicates that some species feed on and are attracted to fungi, such as *Geotrichum candidum*. In the present study, as part of our continuing effort to characterize mechanisms of earthworm chemosensation, we tested whether ethyl hexanoate and ethyl pentanoate, two compounds produced by *G. candidum,* are appetitive to the European nightcrawler (*Dendrobaena veneta*)*.* In a soil T-maze, both of these compounds significantly repelled individual earthworms in a dosage-dependent manner, this result ran counter to our initial hypothesis. *D. veneta* also avoided ethyl hexanoate and ethyl pentanoate in an assay we specifically developed to test an earthworms aversion to chemical stimuli in soil. In both of these assays, ethyl hexanoate was aversive at lower concentrations than ethyl pentanoate. These findings further clarify our understanding of the chemical cues that trigger the decision of *D. veneta* to select a particular soil-environment, and emphasize that different earthworm species may react very differently to commonly encountered chemical stimuli.

## Introduction

The chemical senses of earthworms have long been a subject of speculation and study (Darwin, 1881; Magnus, Kenneth & Resnick, 1999; Rota, 2011). However, most studies of earthworms have an ecological reference frame (Stürzenbaum et al., 2009). This approach has established the significant positive (Aira & Piearce, 2009; Agapit et al., 2018) and negative (Craven et al., 2017) effects earthworms have on soil properties, structure, and processes (Sharma, Tomar & Chakraborty, 2017; Fierer, 2019). Earthworms can change the physicochemical characteristics of the soil or influence resident organisms (Bohlen et al., 2004; Ferlian et al., 2018; McCay & Scull, 2019). These types of studies also describe how earthworms respond to polluted soil in aggregate (Shi, Tang & Wang, 2017) and the International Organization for Standardization even describes a bioassay utilizing groups of earthworms to determine soil quality (ISO, 2017). However, little is known about the mechanisms determining how earthworms make the decision of what soil they will live in and consume (Curry & Schmidt, 2007).

Our current understanding of earthworm chemical senses is primarily based on anatomical studies, and on the chemicals used as vermifuge. Several studies have demonstrated that allyl isothiocyanate (AITC) and other compounds are repellent to a variety of earthworm species in the field (Pelosi et al., 2009; Pelosi et al., 2014; Singh, Singh & Vig, 2016; Singh et al., 2018; Silver et al., 2019). However, these chemicals were likely aversive due to the stimulation of polymodal nociceptors (*i.e.,* “pain fibers”), which mediate the sense of chemesthesis (Richards, Saunders & Silver, 2010; Saunders & Silver, 2016), rather than stimulation of the gustatory or olfactory systems in earthworms. Purported chemosensory organs are concentrated on the head and prostomium, and found on every segment in multiple species (Knapp & Mill, 1971; Kiszler et al., 2012), but no study has ever demonstrated that these organs are, in fact, capable of detecting any chemicals. Electrophysiological techniques have shown that the segmental nerves innervating earthworm skin respond to diverse compounds including acids, NaCl, quinine, and sugar (Laverack, 1960; Laverack, 1961; Knapp & Mill, 1968). However, this only demonstrates that the chemicals are detected, not the identity of the cellular detectors, or if an earthworm finds a particular chemical appetitive or aversive. We have little understanding of the mechanism which trigger these animals to make choices about their food (Curry & Schmidt, 2007), and even less understanding of the specific receptors and their chemical ligands which might mediate feeding behavior of earthworms.

We are interested in characterizing earthworm chemical senses using modern neuroscience methods, and while our preliminary experiments have established the European nightcrawler (*Dendrobaena veneta*, previous *Eisenia hortensis*) as a feasible model species, the main prerequisite for achieving this goal is determining the compounds that elicit stereotyped behaviors. Upon encountering a study that identified ethyl pentanoate and ethyl hexanoate as appetitive to the *Eisenia fetida* (Zirbes et al., 2011), we hypothesized that *D. veneta* may also be attracted to these compounds and tested this premise using T-maze and burrowing assays.

## Materials & Methods

European nightcrawlers, *D. veneta*, were purchased from Uncle Jim’s Worm Farm (Spring Grove, PA, https://unclejimswormfarm.com). We regularly examined the morphology of the prostomium and seta with scanning electron microscopy and conducted DNA barcoding (Pop, Wink & Pop, 2003) to ensure that our worms were *D. veneta.* Earthworms were housed in plastic tubs (45 L) containing topsoil, in a room on a 12–12 light-dark cycle and 6 g of Purina worm chow (Gray Summit, Missouri, USA) and 400 ml of deionized water were added to the tubs twice a week. 24 h before experiments, we selected earthworms with a visible clitellum and starved them in a beaker lined with moist filter paper. Each worm was used only once; after experiments the worms were sacrificed by freezing and disposed of with biohazardous waste.

All topsoil (Timberline Top Soil, Oldcastle lawn & Garden Inc, Atlanta, Georgia, USA) was sieved (60 mm aperture) and dried at 30 °C for 48 h before use. To ensure that the soil had consistent moisture across all experiments, we added deionized water to dry soil at a ratio of 20 ml for every 80 ml of soil and mixed until homogeneous. The only difference in the soil between experiments was the amount of ethyl hexanoate (EH, Aldrich) or ethyl pentanoate (EP, Oakwood Chemicals) added to the water. Soil pH was within 4.9 and 5.1 for all conditions (Kelway Soil pH and Moisture Meter, Kel Instruments Company).

**Figure 1:**
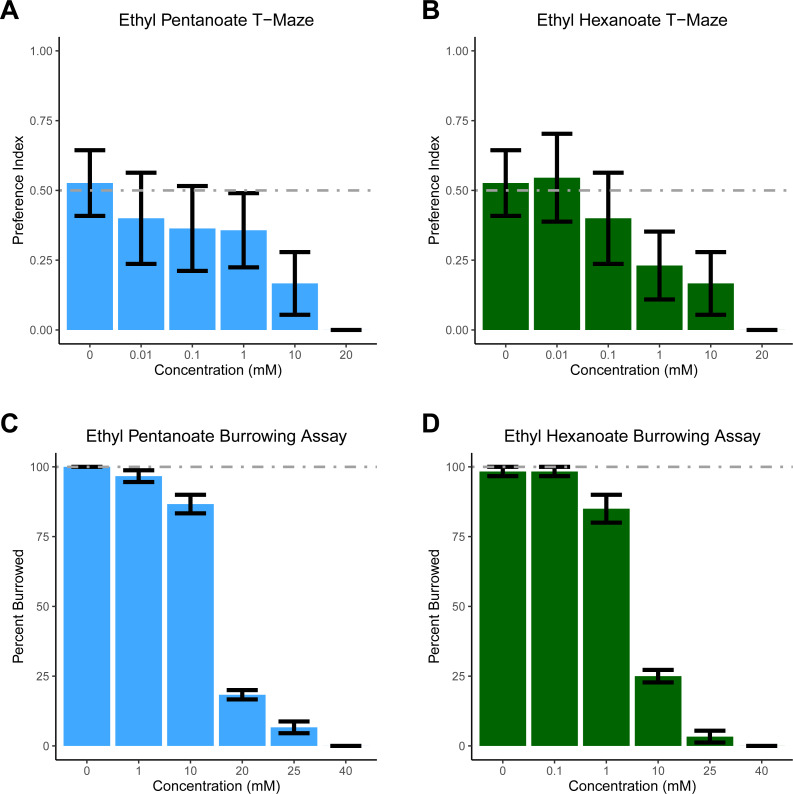
Dendrobaena veneta avoids Ethyl Pentanoate and Ethyl Hexanoate. In a T-Maze assay, *D. veneta* avoids (A) Ethyl Pentanoate (light blue) and (B) Ethyl Hexanoate (dark green) in a dosage-dependent manner when given a choice between these chemicals and control soil. Similarly, in a burrowing assay, fewer *D. veneta* burrow in soil as concentrations of (C) Ethyl Pentanoate and (D) Ethyl Hexanoate increase. Dashed lines depict the expected control values. Bars represent mean ± SEM.

For T-maze experiments, two 80 ml batches of soil were made (one containing either EH or EP, the other containing only water); this amount of soil was sufficient to fill 10 T-mazes. The T-maze was described previously (Silver et al., 2019). One arm of the maze contained soil and water, while the other contained soil treated with increasing concentrations of EP and EH. All experiments were conducted in the dark with a light shining above the T-maze to elicit burrowing (Doolittle, 1972). One *D. veneta* was placed head-first into the top opening of the T-maze and after 10 min, the position of the earthworm’s head was recorded: 0 if the head was on the vehicle side to indicate avoidance of the stimulus; 1 if the head was on the stimulus side to indicate the worm was attracted to the experimental substance; and if a worm failed to burrow it was not scored. Only 7.8% of the 165 worms tested in the T-maze failed to burrow. Each concentration of EP and EH was tested on 10–19 individual worms. To generate a preference index, all the scored replicates were averaged (Fig. 1AB).

For the burrowing assay, soil was made in 800 ml batches; this amount of soil was divided equally between ten 3 oz plastic cups (KCH Corporation, Brooklyn), in a lighted room. We placed a single earthworm on top of the soil in each cup, ensuring that the body of the earthworm did not contact the edge of the cup—in developing this assay we observed that if the worms touched the plastic cup they almost always crawled out. After 10 min, the position of the worm’s head was coded 1 if it was burrowed into the soil or 0 if it was on top of the soil or had crawled out of the cup. These experiments were conducted in batches of 10 and each batch counted as a replicate; each concentration of EP and EH was tested on 6 replicates, and worms were not reused between groups (*i.e.,* for each concentration 6 groups of 10 worms, or 60 total worms were tested.

Due to the T-maze measuring a discrete either-or outcome, we chose to model earthworm behavior with a binominal regression where concentration was an independent predictor. This was accomplished by wrapping the output of the functions glm(family = “binomial”) from the stats package to the summary() function from the base package in R version 4.0.1. As we designed the output of the burrowing assay to produce continuous data (each data point could range from 0 to 10, as 10 worms were tested in each replicate), we were able to analyze it with the function aov(), wrapped in anova() from the stats package (R Core Team, 2020).

## Results

Based on previously published experiments with EH, EP, and a closely related worm species *E. fetida*, we had initially hypothesized that both compounds would attract *D. veneta* (Zirbes et al., 2011). However, as concentration increased, significantly fewer earthworms chose the arm treated with both EP (glm *p* = 0.003) and EH (glm *p* = 0.002). Earthworms did not show a side preference when both sides of the T-maze contained only soil, ∼50% of the earthworms chose each arm of the T-maze (Fig. 1AB; 0 mM EP and EH). At concentrations of 20 mM, all the earthworms tested avoided both EP and EH in the T-maze. These data suggest that both EP and EH are repellent to *D. veneta*.

To ensure that our results were not an artifact of the T-maze paradigm, we tested EH and EP in a second assay designed specifically to assess aversion. In the burrowing assay, all but one of the 120 earthworms we tested in control conditions burrowed (Fig. 1CD, 0 mM EP and EH). In contrast to these control experiments and consistent with the T-maze assay, increasing concentrations of both EP (Figs. 1C, F(1,34) =129.83, *p* = 3.724 ×10^−13^) and EH (Fig. 1D, F(1,34) =213.55, *p* = 3.206 × 10^−16^) significantly reduced the number of burrowing earthworms. Additionally, the burrowing assay also revealed that *D. veneta* avoided EH at lower concentrations than EP, with the majority of earthworms burrowing in 10 mM EP but 20 mM EP and 10 mM EH producing similar levels of pronounced avoidance. Taken together, these results also suggest that EP and EH are both repellent to *D. veneta* and that EH is more aversive than EP at the same concentration.

## Discussion

The results from the T-maze assay were surprising and stand in contrast to the report indicating that EP and EH attracted *E. fetida* (Zirbes et al., 2011). Due to this unexpected result, we felt we should test these compounds in an assay, which specifically tests aversion to ensure our result was not an artifact of the T-maze assay. Thus, we examined the effect of EP and EH on *D. veneta* in a new behavioral assay developed to examine earthworm responses to potentially repellant compounds. In this assay, earthworms are placed on soil in a cup containing the chemical of interest. Since it is the natural tendency of earthworms to burrow into soil, logic would suggest that they would **not** burrow only if something about the soil is aversive. In contrast to the T-maze assay, higher concentrations were required to prevent 100% of the earthworms tested in the cup assay from burrowing in soil (Fig. 1CD, 40 mM). Since worms show an overwhelming preference for burrowing in untreated soil (Fig. 1CD, 0 mM), the most likely interpretation of the difference between the two assays is that worms will tolerate some amount of EH and EP if the only soil present contains those chemicals. Thus, for the earthworm being burrowed has a positive hedonic valence, which must be overcome by a negative stimulus before the worms will avoid the soil.

A large number of publications exist on the feeding ecology of earthworms (see Curry & Schmidt, 2007 for a review (Curry & Schmidt, 2007)) and different species earthworms exhibit food preferences for different materials. For example, *L. terrestris* prefers material that has been inoculated with specific microorganisms (Wright, 1972; Cooke, 1983). Different earthworm species also have preferences for specific fungi (Moody et al., 1995). *E. fetida* is attracted to the fungus *Geotrichum candidum* and specifically two chemicals it produces, ethyl pentanoate and ethyl hexanoate (Zirbes et al., 2011). Since *E. fetida* and *D. veneta* both are epigeic earthworms with similar ranges that feed primarily on plant litter, the species are likely to encounter similar chemical signals. Taken together with the assertion that many earthworms are fungal-feeding decomposers that show preferences for particular fungi (Curry & Schmidt, 2007), we tested the hypothesis that *D. veneta* and *E. fetida* would respond similarly to both EH and EP as they indicate the presence of a potential food source, *G. candidum*. Not only was *D. veneta* not attracted to EP and EH, but that earthworm species also found the two compounds repellent.

Genetic differences between *E. fetida* and *D. venta* could explain the observed behavioral differences. Related species can exhibit vastly different taste preferences. In the mammalian lineage, mice and rats or even different strains of mice can exhibit differential preferences for the same tastant (Sclafani, Vural & Ackroff, 2017). Indeed, studies on human twins have demonstrated that differences as subtle as single nucleotide polymorphisms in chemosensory associated genes can correlate with differences in the perception of taste and smell (Knaapila et al., 2012). If the sequence identity of chemosenory receptors differ between *E. fetida* and *D. veneta*, those differences could underlay differing perceptions of the same compounds. Additionally, even if the sequence identity of the chemosensory receptors is very similar, expression of a receptor gene by a different population of neurons could result in the same chemical triggering opposite responses. Unfortunately, a prerequisite for distinguishing between these possibilities requires identifying of the molecular mechanism of earthworm chemoreception that are currently unknown.

An alternate interpretation of our results is that the addition of any chemical to the soil alters its physical properties, and *D. veneta* is responding to those changes *via* somatosensation. However, we have observed *D. veneta* burrowing rates of over 95% in 20 mM capsaicin, 1 M sucrose and 1 M sorbitol in this assay (Smith et al. 2018; Saunders et al., 2020). The possibility still exists that the avoidance we observed is due to physical changes specific to organic esters, like EP and EH, but 1 M sucrose noticeably alters the physical properties of the soil more than 40 mM EP or EH and has no effect on burrowing.

Another possible explanation for the differences between this study and Zirbes et al. is the different assays used. In the current study, the chemicals were diluted to a known concentration and mixed with a fixed volume of dried soil. However, one confound of this approach is the earthworms are in direct contact with the chemical solution, resulting in exposure to a much higher concentration of EH and EP than if it were vaporized. This raises the possibility that all the concentrations of EH and EP tested were too concentrated to trigger appetitive behavior. Even so, we believe this possibility to be unlikely, as over 80% of *D. venta* burrowed in 10 mM EH (Fig. 1C), but concentrations several orders of magnitude lower failed to attract *D. venta* in the T-maze assay (Fig. 1C). Similarly, over 80% of *D. venta* burrowed in 1 mM EP (Fig. 1B), but did not find comparatively lower concentrations attractive in the T-maze (Fig. 1D). Additionally, any limitations that come from having direct contact with the chemical stimulus are balanced by the ability to precisely control chemical concentration, allowing it to be examined as an experimental factor.

In contrast, the “soil olfactometers” designed by Zirbes et al. (2011) relied on pure EH and EP or *G. candidum* filtrate placed on filter paper, which would then diffuse through the soil. A strength of this approach is that it most closely related to the natural environment where fungi would likely be found growing in patches of high concentration and that groups of earthworms would be present. However, as we are interested in what molecular and neural mechanisms underlie earthworm behavior, a more naturalistic assay also carries with it more confounds. For example, the behavior of earthworms in groups is likely influenced by conspecific chemical signals (Rosenkoetter & Boice, 1975) or touch (Zirbes et al., 2010). It is possible that the presence of other earthworms altered the hedonic valence of EP and EH. In contrast, we have argued for testing earthworms individually to avoid this confound, specifically when studying earthworm chemosensation (Silver et al., 2019). In the T-maze and burrowing assay, 100% avoidance was achieved at different concentrations of EH and EP. Taken together, these details suggest that earthworms, like most animals, are integrating multiple sensory stimuli to calculate the overall hedonic value of a decision’s outcome. The implication being that naturalistic assays that are ideal for addressing ecological hypotheses are not well suited for addressing questions regarding the behavioral neuroscience of earthworms, and vice versa.

Many studies describe the effect chemicals have on earthworm biology in ecological paradigms (Shi, Tang & Wang, 2017). Much of this literature examines how earthworms react to soil pollutants and, in some cases, can sequester these chemicals from the soil (Pattnaik & Reddy, 2011; Shi, Tang & Wang, 2017). Earthworms wield significant positive effects on soil ecology and can encourage the growth of specific plant species. Understanding the hedonic valance of particular chemical signals to earthworms can generate novel approaches for how we use earthworms to manage soil quality. It is within the realm of reasonable possibility that chemical signals exist, which could attract earthworms to normally noxious polluted soil, encouraging them to sequester pollutants and rehabilitate the soil ecosystem. Conversely, there are some ecosystems in which earthworms are an invasive and destructive species. Progressively warm temperatures have allowed earthworms to enter and destroy boreal forests (Fierer, 2019). In these cases, it might be able to use chemical signals that are specifically aversive to earthworms to protect these ecosystems. All the possibilities are predicated on a better understanding of earthworm chemical senses on the behavioral, systems and molecular level.

We have very little information about the earthworm sensory systems and associated genes reasonable for the detection of the food they eat or the avoidance of potentially harmful substances. There are at least two possible sensory structures in the epidermis of earthworms–the epidermal sensory organs and the solitary sensory cells (Knapp & Mill, 1971)—which could be chemosensory. Nerves fibers arising from near these structures appear to respond to chemical stimuli (NaCl, HCl, NaOH, quinine hydrochloride, sucrose, glucose, glycerol) (Laverack, 1960; Laverack, 1961). But these responses have not yet been attributed to any specific type of sensory cell in the epidermis. It is not even known if earthworms chemoreceptors are more closely related to the ionotropic arthropod gustatory receptors (Gr) and odorant receptors (Or) or the traditional G-protein coupled metabotropic receptors subfamilies responsible for taste (Tas1R and TasR2) and olfaction (ORs) in the vertebrates lineage (Montell, 2009; Robertson, 2009; Yarmolinsky, Zuker & Ryba, 2009). EP and EH are both organic esters, a class of compounds which are well known to stimulate odorant and olfactory receptors, but neither of these compounds are structurally similar to any of the compounds which reportedly stimulate earthworm sensory nerves.

## Conclusions

To help elucidate the neural and molecular mechanisms of chemoreception in earthworms, we have begun developing behavioral assays to assess an earthworm’s repellant and appetitive responses to specific chemicals. Previously, we reported using a T-maze assay to demonstrate concentration-dependent responses to three different irritants, which are known to stimulate Transient Receptor Potential (TRP) channels (Silver et al., 2019). In the present study, we tested a presumably appetitive stimuli using this T-maze assay and developed another assay to quantify aversion for earthworms. In these assays, *Dendrobaena veneta* avoided both ethyl hexanoate and ethyl pentanoate in a dosage-dependent manner.

##  Supplemental Information

10.7717/peerj.12148/supp-1 Supplemental Information 1 Raw data of *D. veneta*’s response to ethyl pentanoate and ethyl hexanoate in T-maze and burrowing assays. The raw data in a “tidy” format for the T-maze and burrowing assays are stored in worksheets titled “Tmaze” and “burrowing”. Each line represents a single replicate. Columns are as follows: **Compound** is the name of the chemical tested; **Concentration.mM** is the millimolar (mM) concentration of the chemical tested; **head. location** is the location of the worm’s head at the end of the T-maze assay; **score** is the numerical score assigned to each replicate in the T-maze based on the location of the worms head; **number.worms** is the number of worms tested in each burrowing assay replicate, always 10 for these experiments; **number.burrowed** is the number of worms whose head was under the soil at the end of each burrowing assay replicate; **number.notBurrowed** is the number of worms whose head was not under the soil at the end of each burrowing assay replicate.

## Additional Information and Declarations

### Data Availability

The following information was supplied regarding data availability: Raw behavioral outputs from experiments are available in the Supplementary File.
